# Recognition of Broken Wire Rope Based on Remanence using EEMD and Wavelet Methods

**DOI:** 10.3390/s18041110

**Published:** 2018-04-05

**Authors:** Juwei Zhang, Pengbo Zheng, Xiaojiang Tan

**Affiliations:** 1 College of Electrical Engineering, Henan University of Science and Technology, Luoyang 471023, China; 150304210236@stu.haust.edu.cn; 2 Power Electronics Device and System Engineering Laboratory of Henan, Henan University of Science and Technology, Luoyang 471023, China

**Keywords:** ensemble empirical mode decomposition, wavelet denoising, super-resolution reconstruction, non-destructive testing

## Abstract

The magnetic flux leakage method is widely used for non-destructive testing in wire rope applications. A non-destructive testing device for wire rope based on remanence was designed to solve the problems of large volume, low accuracy, and complex operations seen in traditional devices. A wavelet denoising method based on ensemble empirical mode decomposition was proposed to reduce the system noise in broken wire rope testing. After extracting the defects image, the wavelet super-resolution reconstruction technique was adopted to improve the resolution of defect grayscale. A back propagation neural network was designed to classify defects by the feature vectors of area, rectangle, stretch length, and seven invariant moments. The experimental results show that the device was not only highly precise and sensitive, but also easy to operate; noise is effectively suppressed by the proposed filtering algorithm, and broken wires are classified by the network.

## 1. Introduction

Wire ropes are structural components made of twisted wire that are widely employed in diverse areas. The safe usage of wire ropes is directly related to the production lifetime and personnel safety. Therefore, it is of great significance to develop an online detection and quantitative inspection system for wire ropes [1]. ElectroMagnetic Testing (EMT), the advantages of which include low cost, high reliability, and suitability for online detection of wire rope, has been widely used in wire rope application. EMTs include eddy current testing, magnetic particle testing, Magnetic Flux Leakage (MFL) detection, magnetic memory detection, microwave detection, and other methods. Among these, the MFL method can detect the surface and internal defects of wire rope, and has been greatly developed for its simple structure and portability [2].

In a traditional MFL detection, wire rope is magnetized to saturation, after which a magnetic probe is used to measure the MFL distribution. According to the form of the magnetic field source, there are two techniques: coil magnetization [3,4] and permanent magnet magnetization [5,6,7,8,9,10]. Singh [3] designed a magnetized device consisting of a variable-current saddle-shaped coil. This apparatus can be adjusted by controlling the magnitude of the current, but the device cannot be continuously used for the massive heating of the coil. Jomdecha et al. [4] improved a coil device of solenoid structure instead of traditional coil magnetizer. The magnetic field strength can be adjusted by changing the magnetizing current or magnetizing coil number. The multiple symmetrical yoke structures were composed of circumferentially distributed wire rope to magnetize its uniform saturation [5,6,7,8]. Wang et al. [9] considered the effects of different lift-off distances on detection accuracy during the magnetization process, and proposed an improved magnetization device. Xu et al. [11] used the finite element analysis method to optimize the structure of the excitation device, which was then validated by the experiment.

In a testing system, the magnetic field is converted into an electrical signal by a magnetic-to-electric convertor, such as an induction coil, a fluxgate, a Hall element, or a magnetoresistive sensor. Cao [12] proposed a detection device based on a Printed Circuit Board (PCB) split differential coil, obtaining a sum of MFL circumferential distribution signals. The device is useful for a certain span in the axial direction of the wire rope, but it is not sensitive to small width defects and circumferential defect information. Jomdecha et al. [4] improved the traditional induction coil by designing an induction coil array, with coils arranged on the circumferential wire rope. This system solved the problem of MFL circumferential information loss. Zhao et al. [13] designed a detection device in which 30 Hall sensors evenly surround the circumferential wire rope. This device can effectively obtain the information of the circumferential MFL distribution, but the Signal-to-Noise Ratio (SNR) of the collected signal is low. Peterka et al. [14] presented the results obtained by tracking the magnetic flux around the cable end and the signal runs from a particular design. Additionally, they investigated scanning elements placed above artificial defects created close to the cable end.

The signal that is collected by magnetic sensors contains ample background noise, so it is necessary to filter system noise. Cao et al. [12] proposed an algorithm for adaptive parameter spatial notch filtering to suppress strand wave, and the wavelet packet was used to filter out the high-frequency random noise. Zhang et al. [15,16] used the wavelet based on compressed sensing to denoise the strand wave and high-frequency noise, and then further proposed a channel-balance method based on the Hilbert-Huang transformation. Zhang et al. [17] used a spatial filter to reduce noise and smoothen the defect image. Zhang et al. [18] pretreated the MFL grayscale and effectively suppressed the noise interference. Tian et al. [19] combined the wavelet transform and the morphological transform to create a morphological filter algorithm that suppressed the interference associated with the baseline drift in the wire rope signal.

There are some problems among the existing MFL methods for defect detection in wire ropes:the excitation devices are cumbersome and inconvenient, the defect cannot be positioned in the circumferential direction, the wire rope is magnetized unevenly, and the SNR is low. A device to detect wire rope surface remanence strength was designed to solve these problems, and this solution is described in this paper. The wire rope was magnetized by permanent magnets, and the MFL information of the wire rope surface was collected after magnetization with giant magnetoresistive sensors, arranged evenly around the circumference of the wire rope. A wavelet filtering method based on Ensemble Empirical Mode Decomposition (EEMD) was used to denoise the original signal. The two-dimensional defect signal was processed and analyzed by digital image technology. To achieve quantitative classification, the defect image characteristics were extracted to express the MFL distribution information. The processing data were normalized to obtain the MFL grayscale image. The cubic spline interpolation was used to improve the circumferential resolution. The method of modulus maxima was used to locate and segment defects from the MFL image. The wavelet super-resolution reconstruction method was used to improve the resolution of the segmented image. Image descriptions of area, rectangle, elongation, and seven invariant moments were extracted as the feature vector of the defect, which was the input of a Back Propagation (BP) neural network, used to classify the defects.

## 2. Remanence Information Collection

### 2.1. Data Collection Platform

**Figure 1:**
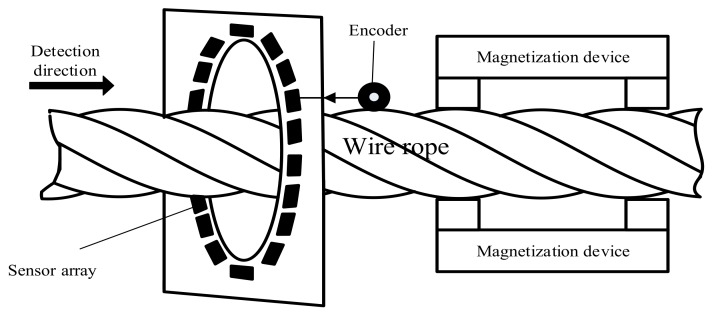
Schematic of data collection.

A data acquisition platform is designed in this section, and as shown in Figure 1, the system mainly includes an excitation source, sensor array, pulse generator, and control system which consisted of a control core, data storage, a signal adjustment, and an analog-to-digital converter.

**Figure 2:**
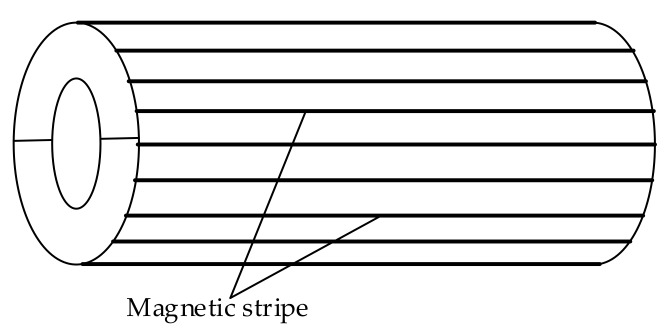
Schematic of excitation device.

**Figure 3:**
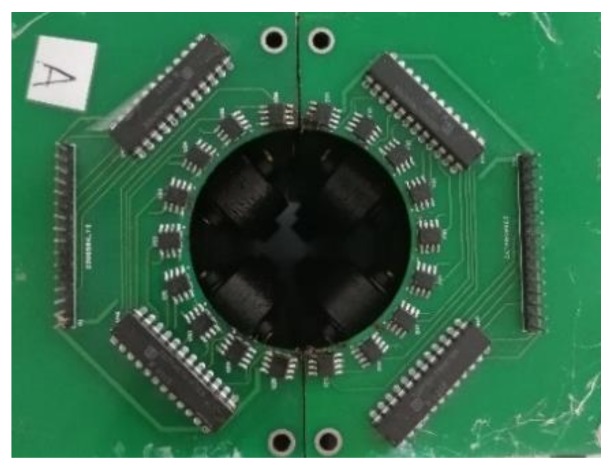
Sensor array.

The excitation device consisted of magnets, the structure of which is shown in Figure 2. A plurality of elongated permanent magnets was uniformly distributed around the wire rope circumference; the value of the field generated by the magnetizing device was 50 mT. In traditional MFL detection, the commonly used sensors are coil sensors, hall sensors, tunnel reluctance sensors, flux gates, and so on. These are either low precision sensors or they are too bulky to integrate. A Giant Magneto Resistance (GMR) sensor has the advantages of high sensitivity, easy integration, and low energy consumption; considering cost and performance, it is a suitable sensor for the measurement of this paper. The sensor array was designed as shown in Figure 3, and 18 GMR sensors evenly surround the wire rope circumference, their sensitive surfaces facing the rope wire. The magnetic induction linear range of the GMR sensor used in the detection board was 0.6–3 Oe (Gauss in air). In order to ensure that the sampling was spatially equidistant, a photoelectric encoder was used to trigger data acquisition. An STM32F407 chip was used as the control chip. In addition, the 12-bit Analog-to-Digital Convertor (ADC) converted analog signals into digital ones, since the relative error of the 12-bit ADC was 0.37% and was hence insufficiently accurate.

### 2.2. Data Collection

The acquisition processes of the remanence detection method were as follows: wire rope was magnetized by the magnetizing device (this process was usually repeated two to three times), the sensor array and encoder were connected to the control board, and the connected device was loaded onto the magnetized wire rope, which was moved along the wire rope from any end when the encoder rotated synchronously. According to the encoder pulse signal, the control board collected the data from 18 channels. Once the rope was magnetized, the rope could be detected the following week without magnetization. In this experiment, the types of broken wire included one, two, three, four, five, and seven broken wires.

**Figure 4:**
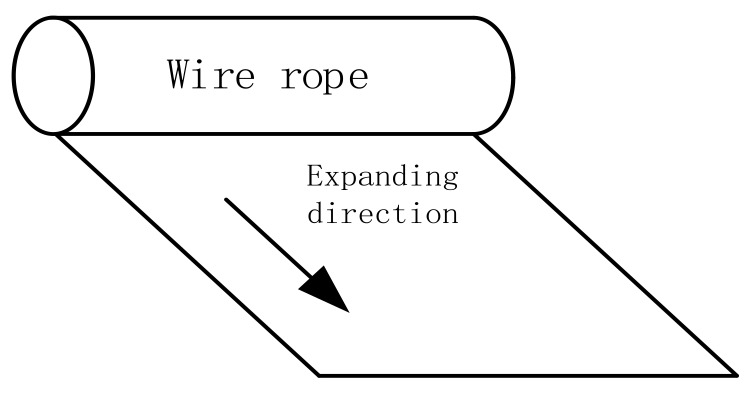
Expanding in circumferential direction.

**Figure 5:**
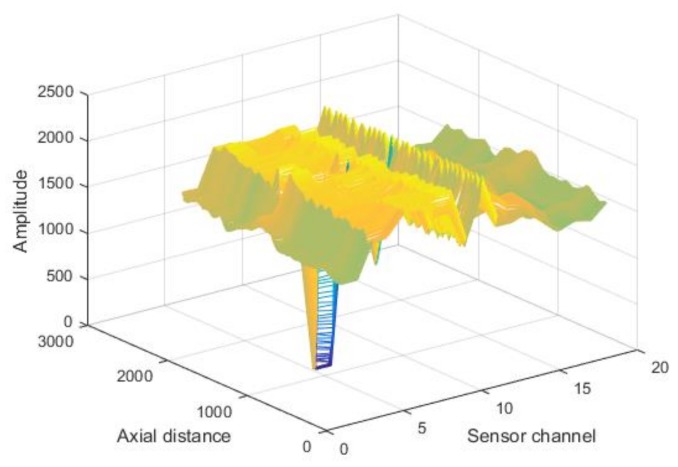
Schematic of original data.

As shown in Figure 4, the experimental data were expanded along the circumferential direction, with the first channel being the starting point. A two-dimensional array of M×N resolution was obtained, where M denotes the 18 sensor channels and N is the number of sampling pulses. The magnetic field intensity without defects is about 0.78 Oe, and the magnetic field intensity of a common defect is about 2.97 Oe. Parts of original data are shown in Figure 5.

## 3. Data Processing

As shown in Figure 5, the original data contained a significant amount of background noise. In this section, a wavelet-filtering algorithm based on EEMD is proposed to suppress system noise.

### 3.1. EEMD Theory

Empirical Mode Decomposition (EMD) is a nonlinear signal analysis method that decomposes the signal into several characteristic functions by the time-domain characteristics of the signal. This method can decompose the signal into a series of single component signals. Each component contains only one oscillation mode, which are collectively called Intrinsic Modal Functions (IMFs) [20]. An IMF should meet the following conditions:

(1) For an IMF component, the number of its maxima and minima is equivalent to 0 crossings, or they differ by 1 at most.
(2) The average of the maxima and minima, as defined by the envelope, should be 0 at any given moment.

However, when implementing the EMD algorithm, modal aliasing and boundary effects will occur between the IMFs; in order to avoid these problems, Huang improved the traditional EMD with EEMD [21]. The EEMD decomposition principle is as follows: Wu et al. [22] pointed out that the EMD of white noise is equivalent to a binary filter group. When the additional white noise is evenly distributed through the whole time-frequency space, this time-frequency space is separated into different scale components by the binary filter group. EEMD differs from EMD containing white noise in terms of signal and computing average of same-scale IMFs [23].

### 3.2. Wavelet Theory

Wavelets comprise a time-frequency multiresolution analysis technology that is widely used for non-stationary signals. Most signals are non-stationary; that is, their frequency changes with time. The changes are both fast and slow: fast change corresponds to the high-frequency part that represents the signal details, and the slow change corresponds to the low-frequency part that describes the signal profile.

In order to implement fast decomposition, Mallat [20] proposed a pyramid wavelet decomposition and reconstruction algorithm to achieve multi-resolution analysis, which is called the Mallat algorithm. The basic idea of this algorithm is that an approximation $H_{j}f$ of an energy-limited signal $f\in L^{2}(R)$ is resolved under the resolution $2^{j}$, of which the approximation $H_{j-1}f$ in the resolution $2^{j-1}$ is obtained by a low-pass filter, and the detail $D_{j-1}f$ is obtained by a high-pass filter. The following formulas reflect decomposition and reconstruction:

(1) $$\begin{array}{l} a_{j+1}\left(k\right)=\sum_{n=-\infty }^{\infty }a_{j}(n)h_{0}(n-2k)=a_{j}(k)*{\overset{-}{h}}_{0}(2k)\\ d_{j+1}\left(k\right)=\sum_{n=-\infty }^{\infty }a_{j}(n)h_{1}(n-2k)=a_{j}(k)*{\overset{-}{h}}_{1}(2k) \end{array}$$

(2) $$a_{j}(k)=\sum_{n=-\infty }^{\infty }a_{j+1}(n)h_{0}(k-2n)+\sum_{n=-\infty }^{\infty }d_{j+1}(n)h_{1}(k-2n)$$

where $\overset{-}{h}(k)=h(-k)$, $h_{0}$ is a low-pass filter, and $h_{1}$ is a high-pass filter. Equation (1) is the decomposition formula of the Mallat algorithm by which the signal can be decomposed into multi-resolution. Equation (2) is the reconstruction formula by which the decomposed signal can be reconstructed.

### 3.3. Algorithm Description

There are a few disadvantages in wavelet denoising: both the selection of the basis function and threshold function is uncertain, and it is difficult to select the optimal parameters. Moreover, if the noise frequency approximates the frequency of the signal, the useful information is lost. Compared to the wavelet transform, EMD does not require many parameters for a blind signal. The noise is typically among the high-frequency IMF components. Wavelet soft threshold denoising is performed in the IMF components of the defect signal, rather than dealing with the entire signal. This algorithm, to a large extent, overcomes the weaknesses of the wavelet noise reduction method. An analysis of IMF components and remainders can effectively restrain the trend items contained within the signal. In Ref. [24], the wavelet thresholding principle was used in the decomposition modes resulting from applying EMD to a signal, and a similar technique adapted to EMD developed, leading to enhanced denoising performance. Li et al. [25] presented an improved algorithm based on EMD wavelet for electrocardiogram signal denoising, and utilized the adaptability of EMD to make up for the indetermination when choosing a wavelet function. Following this, wavelet adaptive thresholding was performed to prevent the distortion of an EMD algorithm. Hence, the improved EMD wavelet algorithm is efficient and stable in blind signal de-noising.

Combining EMD and wavelet analysis, a wavelet filtering algorithm based on EEMD was designed to remove the system noise. The algorithm is described as follows.

1. EEMD was implemented to the *i*th channel signal *x*_i_:
  (1) The signal $x_{i}$ was extended to obtain the extended signal $\tilde{x_{i}}$;
  (2) The white noise of normal distribution was added to the signal $\tilde{x_{i}}$, resulting in signal $y_{i}$;
  (3) EMD was used to decompose the signal $y_{i}$ to obtain its IMF components;
  (4) Steps (1) and (2) were repeated *k* times, and then *k* groups of IMFs with different white noise were obtained;
  (5) The average of these IMFs was calculated, and each IMF of the signal $x_{i}$ was obtained;
  (6) It was determined whether the termination condition was met; if satisfied, decomposition was stopped. Otherwise, step (3) was repeated to continue the break down.
2. Wavelet soft threshold denoising was used for IMF components which contain a defect signal:
  (1) A db5 wavelet was selected to decompose the IMF with 8-level decomposition;
  (2) The low-frequency coefficient was cleared, and soft threshold quantization was performed by the universal threshold $\sqrt{2\log()}$ for the high-frequency coefficients at each decomposition scale;
  (3) The processing wavelet coefficients *t* were reconstructed by a one-dimensional wavelet reconstruction function, with which the filtered IMF component was obtained.
3. The processed IMF components were superimposed to obtain the clean data.

**Figure 6:**
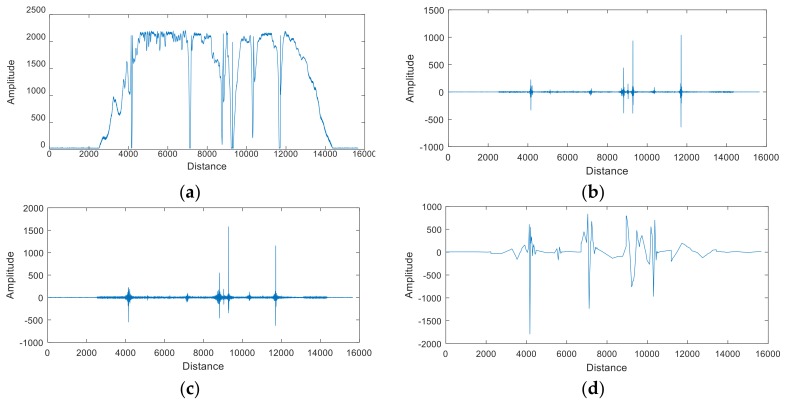

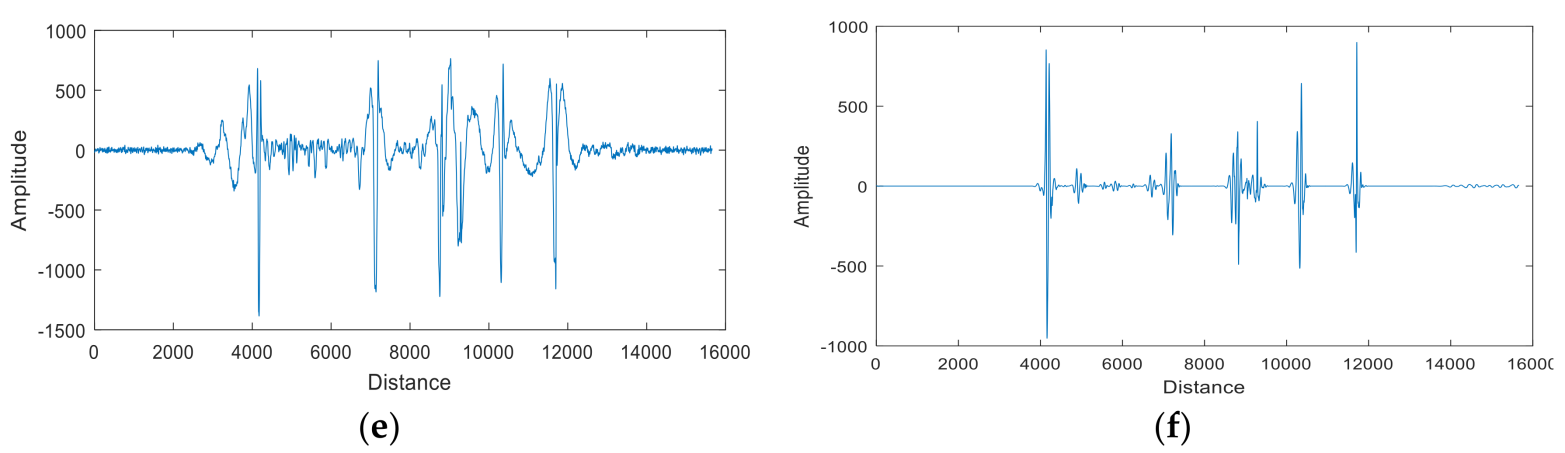
Schematic of single-channel data before and after denoising: (**a**) Single-channel raw signal; (**b**) signal denoised by VMD algorithm; (**c**) signal denoised by EWT algorithm; (**d**) signal denoised by HHT-WFCS algorithm; (**e**) signal denoised by improved EEMD algorithm; (**f**) signal denoised by proposed algorithm.

The data were processed by the proposed algorithm; raw and processed signals of one channel are shown in Figure 6. To evaluate the performance of the proposed algorithm, the SNR is defined as SNR = 20log(*V_s_*/*V_n_*), where *V_s_* is the maximum peak–peak value of the MFL signal, and *V_n_* is the maximum peak-–peak value of the noise. We compared the proposed algorithm with four recent algorithms with the SNR, such as the Variational Mode Decomposition (VMD) algorithm proposed in Ref. [26], the Empirical Wavelet Transform (EWT) algorithm proposed in Ref. [27], the HHT-WFCS algorithm proposed in Ref. [16], and the improved EEMD algorithm proposed in Ref. [28]. The groups of experimental data were included to 15, in order to prove the robustness and effectiveness of the proposed algorithm, as shown in Table 1. It can be seen that the average SNR of the original signal is 17.55 dB, the average SNR of the signal denoised by the VMD algorithm is 44.31 dB, the average SNR of the signal denoised by the EWT algorithm is 42.70 dB, the average SNR of the signal denoised by the HHT-WDCS algorithm is 41.32 dB, and the average SNR of the signal denoised by the improved EEMD algorithm is 42.36 dB. However, implementing the proposed algorithm increases the average SNR to 70.01 dB. The results demonstrate that the proposed algorithm exhibits a good property for the wire rope remanence signal.

**Figure 7:**
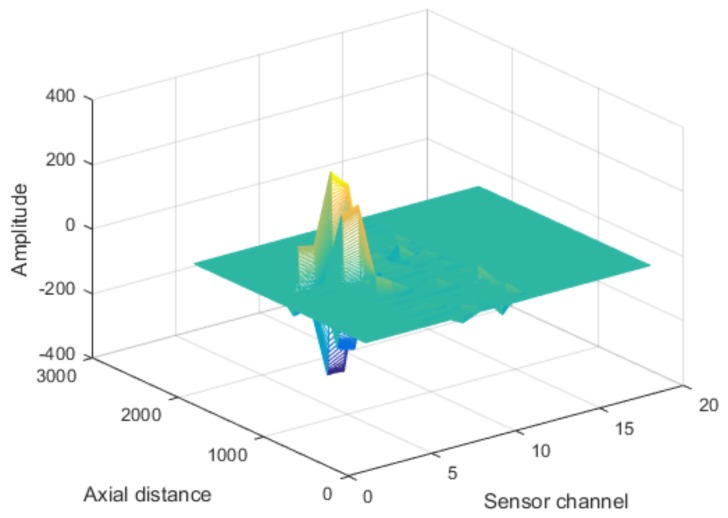
Schematic of filtered data.

The array Leakage Magnetic Field (LMF) data were processed by the wavelet filtering algorithm based on EEMD, where the baseline and various noises could be removed. Figure 7 shows the three-dimensional view of one set of denoising data.

## 4. Magnetic Image Enhancement

**Figure 8:**
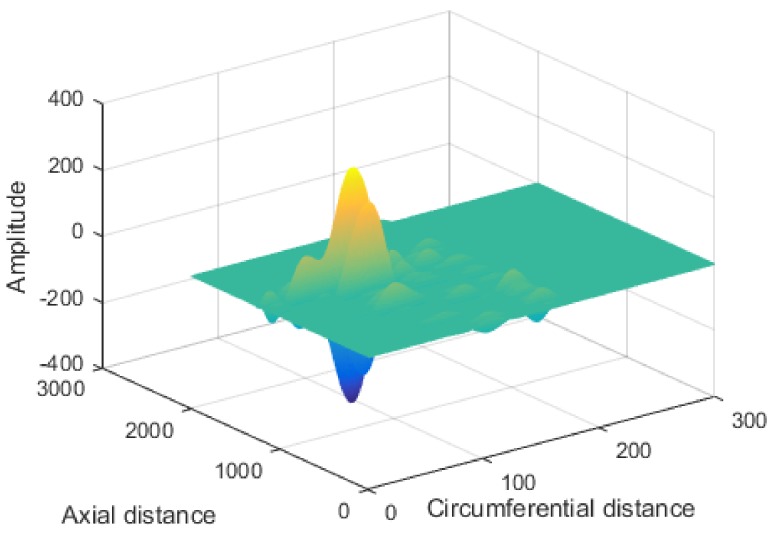
Schematic of interpolation data in circumferential direction.

The defects could easily be extracted and quantitatively identified through image processing technology. The final step was to improve the recognition accuracy. The acquisition system had an 18-channel GMR sensor array; therefore, the circumferential resolution of a magnetic image was only 18, which was much below the axial resolution. To balance the Two-Dimensional (2D) resolution, interpolation was applied to improve the circumferential resolution first. In this paper, cubic spline interpolation was used to interpolate the circumferential points, which were raised to 300, with which the circumferential details of LMF were improved. Figure 8 shows a schematic of part of the interpolated array data.

### 4.1. Normalization and Defect Segmentation

Gray normalization needed to be done after the data interpolation, in which the LMF information was transformed into a grayscale image. In this paper, the LMF data were normalized to (0.255) by the minimum-maximum normalization method. The specific algorithm was used as follows:

(1) The maximum and minimum of the LMF data was found and recorded;
(2) Each piece of LMF data was processed by the following equation:
  (3) $$dat(i,j)=\frac{data(i,j)-\min}{\max-\min}\times 255$$
  where min denotes the minimum of data, max the maximum of data, data the input, and dat the normalized data.
(3) The processed data was converted into 8-bit unsigned integer data and stored.

**Figure 9:**
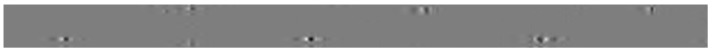
MFL grayscale image.

Following the above three steps, the LMF information is converted into grayscale images. Figure 9 shows the grayscale image of the normalized LMF. Defect positioning consisted of axial and circumferential location. The modulus maxima method was used to locate and segment defects, as follows:

(1) The circumferential average, and a 1D mean signal *d*(*j*) (1 ≤ *j* ≤ *N*), which is the number of sampling points, was calculated.
(2) A threshold was implemented to d(j), where the greater value was retained, and the others were set to 0. Then, d′(j) and position the maximum of d′(j), which is the axial position of the defect, were obtained;
(3) According to defect width, the axial length was approximately 300 pixels, so a 300 × 300 image was segmented along the axial direction;
(4) Along the axial direction, pixels are added to obtain a 1D *a*(*i*) (1 ≤ *i* ≤ 300), then position the maximum of *a*(*i*), which is the defect circumferential location.

After the above processes, the defect could be divided into an image of 300 × 300 resolution.

### 4.2. Wavelet Super-Resolution Reconstruction

**Figure 10:**
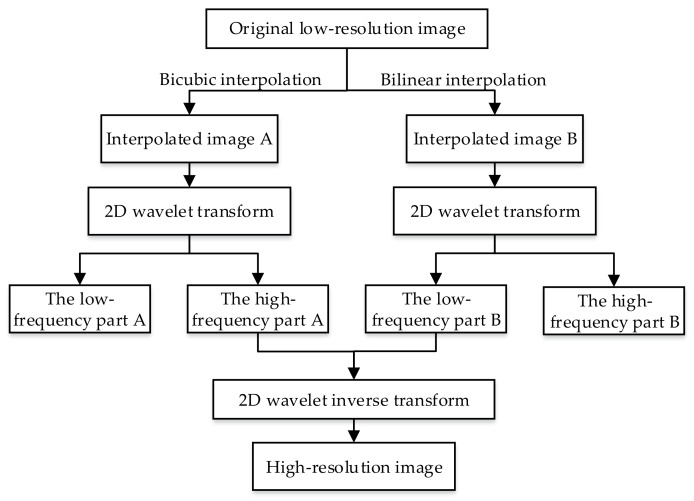
WSR algorithm process.

Wavelet Super-resolution Reconstruction (WSR) can improve the image resolution without losing image details. HR means the image has a high pixel density, and more details can be provided. These details play a key role in quantitative identification. When the resolution is improved, the distance between the characteristics of the sample can be increased, so that the sample can be separated more easily and the accuracy of the classification, improved. The WSR method was used to improve the defect image resolution. WSR is an image enhancement method in which a low-resolution image or image sequence is processed to restore a high-resolution image by computer processing technology. In this paper, WSR was used to improve the resolution of defect grayscale. The WSR algorithm process is shown in Figure 10.

**Figure 11:**
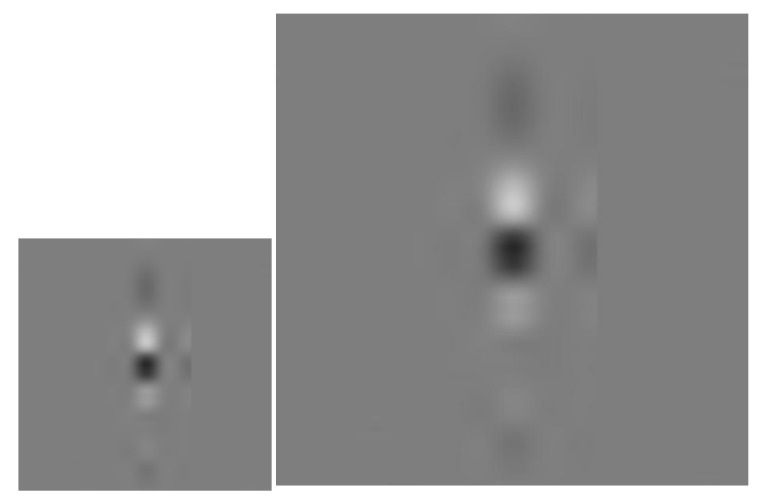
Grayscale before (**left**) and after (**right**) increase in resolution.

As shown in Figure 11, one defect image was reconstructed with this method, and the defect had more obvious edge information with sufficient resolution.

## 5. Quantitative Identification

In this paper, a BP neural network was used to train a classifier that could export the number of broken wires. After extracting the features of a defect image, a three-layer BP neural network was trained to identify the broken wire number.

### 5.1. Feature Extraction

The dimensions of the defect image affect the complexity of the training neural network. Image features are an effective expression of the object that is usually adopted in image pattern recognition because of its excellent dimensional reduction and effective description. The area, squareness, elongation, as well as seven invariant moment features were extracted as eigenvectors of the defect image.

The area *A* is defined as the pixel number of the object region. It reflects the circumference size of the defect and the number of broken wires. The squareness *R* is the ratio of area to product of object length and width. When the *R* value is close to 1, the target area is similar to a rectangle, and as it is close to 0, the region is more complex. *E* is the ratio of width and length. It reflects the shape of the defects and is sensitive to the circular boundary. When the defect shape forms a circle, the short and long diameters are relatively close, and the *E* value is close to 1:

(4) $$A=\sum_{(x,y)\in Q}G$$

(5) $$R=\frac{A}{H\times W}$$

(6) $$E=\frac{\min(H,W)}{\max(H,W)}$$

where *Q* is the defect region, *H* the length of the region, and *W* the width of the region.

The invariant moment features mainly characterize the geometric features of the image area. When the rotation, translation, and scale changes, the invariant moment feature do not change. The 2D p+q moment of a digital image of size M×N is defined as follows:

(7) $$m_{pq}=\sum_{x=0}^{M-1}\sum_{y=0}^{N-1}x^{p}y^{q}f(x,y)$$

where p=0,1,2,⋯ and q=0,1,2,⋯. The corresponding p+q moment is defined as

(8) $$\mu_{pq}=\sum_{x=0}^{M-1}\sum_{y=0}^{N-1}{\left(x-\bar{x}\right)}^{p}{\left(y-\bar{y}\right)}^{q}f(x,y)$$

where p=0,1,2,⋯ and q=0,1,2,⋯, and $\bar{x}=m_{10}/m_{00}$ and $\bar{y}=m_{01}/m_{00}$.

The normalized center moment $\eta_{pq}$ is defined as

(9) $$\eta_{pq}=\mu_{pq}/\mu_{00}^{\gamma }$$

where γ=(p+q)/2+1 and p+q=2,3,⋯.

On the basis of the above definition, seven invariant moments can be deduced as follows:

(10) $$\phi_{1}=\eta_{20}+\eta_{02}$$

(11) $$\phi_{2}={\left(\eta_{20}-\eta_{02}\right)}^{2}+4\eta_{11}^{2}$$

(12) $$\phi_{3}={\left(\eta_{30}-3\eta_{12}\right)}^{2}+{\left(3\eta_{21}-\eta_{03}\right)}^{2}$$

(13) $$\phi_{4}={\left(\eta_{30}+\eta_{12}\right)}^{2}+{\left(\eta_{21}+\eta_{03}\right)}^{2}$$

(14) $$\begin{array}{c} \phi_{5}=(\eta_{30}-3\eta_{12})(\eta_{30}+\eta_{12})[{\left(\eta_{30}+\eta_{12}\right)}^{2}-3{\left(\eta_{12}+\eta_{03}\right)}^{2}]+\\ \left(3\eta_{21}-\eta_{03})(\eta_{21}+\eta_{03})[3{\left(\eta_{30}+\eta_{12}\right)}^{2}-{\left(\eta_{21}+\eta_{03}\right)}^{2}\right] \end{array}$$

(15) $$\phi_{6}=(\eta_{20}-\eta_{02})[{\left(\eta_{30}+\eta_{12}\right)}^{2}-{\left(\eta_{21}+\eta_{03}\right)}^{2}]+4\eta_{11}(\eta_{30}+\eta_{12})(\eta_{21}+\eta_{03})$$

(16) $$\begin{array}{c} \phi_{7}=(3\eta_{21}-\eta_{03})(\eta_{30}+\eta_{12})[{\left(\eta_{30}+\eta_{12}\right)}^{2}-3{\left(\eta_{21}+\eta_{03}\right)}^{2}]+\\ \left(3\eta_{12}-\eta_{03})(\eta_{21}+\eta_{03})[3{\left(\eta_{30}+\eta_{12}\right)}^{2}-{\left(\eta_{21}+\eta_{03}\right)}^{2}\right] \end{array}$$

In this paper, seven invariant moments are substituted for the logarithm absolute values of themselves; the logarithm can narrow the dynamic range, and the absolute value can avoid dealing with the complexity generated by calculating the logarithm of the negative invariant moment. According to the characteristic extraction above, parts of vectors of one wire rope are listed in Table 2.

### 5.2. BP Neural Network

A BP network is a multi-layer feed-forward neural network that is trained according to an error BP algorithm. It is simple and ease of use, and it requires a minimal amount of calculations and parallelism to facilitate its wide application in pattern recognition. BP networks can learn and store a large number of input and output mappings. The topology of a BP neural network includes an input layer, one or more hidden layers, and an output layer. A three-layer BP neural network can approach any non-linear function.

A BP algorithm includes two aspects: forward propagation of signals and back-propagation of errors. In this paper, a BP neural network of 10 × *N* × 7 was designed, where *N* is the number of hidden layer nodes. The network had 10 inputs and seven outputs: the inputs were feature vectors, and the outputs were combinations of 0 or 1 to express the number of broken wires. The hidden layer used “tansig” as the activation function and the output function was “logsig”.

### 5.3. Results Statistics

**Figure 12:**
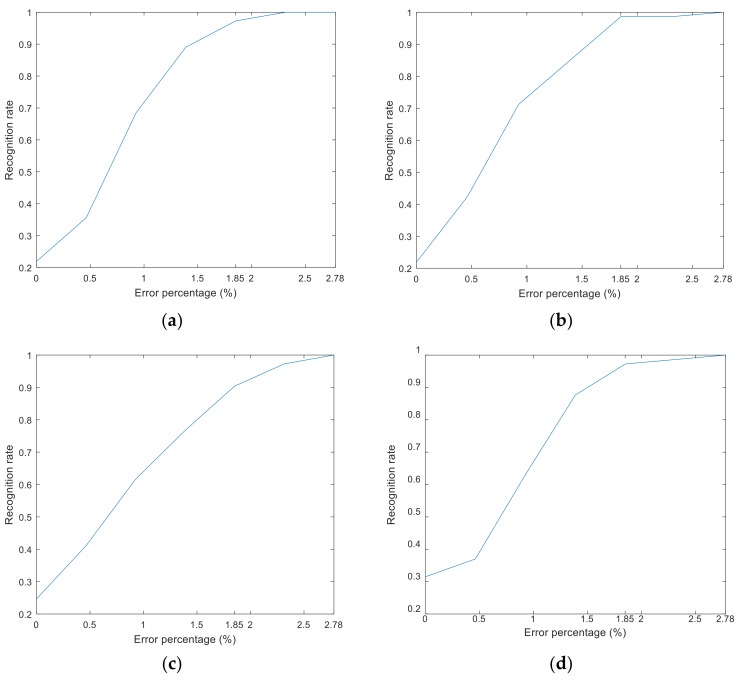
Different numbers of hidden layer node recognition results: hidden layers have (**a**) 15 nodes, (**b**) 17 nodes, (**c**) 21 nodes, and (**d**) 25 nodes.

In this quantitative identification experiment, the diameter of the wire rope was 32 mm, and it contained 216 wires. The distance between the GMR sensors and the wire rope was about 9 mm. It is easy to defect the large broken wires, but it is hard to detect the small broken wires. As long as the detection system can detect the small broken wires, it is easy to detect the large broken wires. It is more meaningful to study the detection of small broken wires. Therefore, a number of 283 standard samples that contained small gaps (approximately 0.2 cm), long widths (1 cm), and sticking wires (approximately 0.2 cm wide and 0.5 cm high) of broken wires from one to seven, were obtained. The neural network toolbox of MATLAB software (MathWorks, Natick, MA, USA) was used to design a BP neural network, and the network was trained to convergence using the trainlm algorithm. The technique of k-fold cross-validation was used to minimize the dependency of the result on the selection of the training and test samples. The samples were divided into four parts: one of them was used as a test sample, and the others were used as training samples; cross validation repeated four times, and the best model was selected. In this paper, a percentage of the number of broken wires was used to create a damage index. This index represents the percentage of broken wires in the total wires and make the damage analysis more intuitive. Figure 12 shows the recognition results of different hidden layer nodes. Through the verification experiment, the recognition accuracy rate was the highest when there were 17 hidden layer nodes. In this condition, the recognition rate reached 98.63% with a limiting error of 1.85%, and the highest recognition error did not exceed 2.78%.

## 6. Conclusions

In this paper, a prototype was designed to acquire MFL distribution, and a GMR sensor array was used as the detection head. Permanent magnets, which were uniformly distributed on a rope wire circumferential surface, were used as the excitation device. The digital data, which were from a 12-bit analog-to-digital convertor through a differential amplification and additive circuit, was stored on an SD card. A wavelet filter algorithm based on EEMD was proposed to reduce system noise, with which the system noise was effectively suppressed. Through signal processing, the defect residual magnetic signal was brighter than that of the original data; here, only defect information was retained. Compared to the filtering algorithm and the algorithm in Ref. [15], the algorithm in this paper has three important advantages: fast calculation speed, good filtering effect, and high SNR. The defect image resolution was enhanced by wavelet SR, by which a high-resolution image was obtained with more defect details. This reconstruction method solved the problem of interpolation leading to loss of image details. Finally, a BP neural network was designed to quantitatively identify defects. The results show that when the allowable error was 1.85%, the recognition rate reached 98.63%, and the highest error did not exceed 2.78%. Compared to the results in Ref. [15], the proposed processing algorithm is more effective, requires less calculation, and yields better recognition accuracy.

The work described in this paper realized wire rope defect localization and quantification, in which the results provided effective evidence for wire rope damage degrees and retirement standards. The feasibility of quantitative detection of wire rope damage based on remanence was verified. The experimental results show that the wavelet filter method based on EEMD could effectively suppress the system noise of the residual MFL signal. Moreover, wavelet SR effectively enhanced the details of defect images and had good applicability. Future research efforts will focus on an optimization of filtering algorithms and quantitative identification.

## Figures and Tables

**Table 1:** The SNR of data denoised by several related algorithms.

Group	Raw Data	VMD Algorithm	EWT Algorithm	HHT-WFCS Algorithm	Improved EEMD Algorithm	Proposed Algorithm
1	12.67 dB	49.99 dB	46.26 dB	37.10 dB	35.30 dB	51.46 dB
2	18.50 dB	51.45 dB	48.40 dB	51.52 dB	45.00 dB	63.62 dB
3	14.53 dB	29.39 dB	23.63 dB	34.19 dB	31.58 dB	74.61 dB
4	17.15 dB	59.01 dB	40.10 dB	46.82 dB	47.44 dB	61.09 dB
5	17.51 dB	49.63 dB	47.78 dB	43.63 dB	42.82 dB	60.24 dB
6	19.31 dB	22.10 dB	21.05 dB	35.78 dB	44.20 dB	80.67 dB
7	20.45 dB	39.38 dB	54.17 dB	45.31 dB	53.59 dB	83.71 dB
8	19.23 dB	43.13 dB	52.93 dB	33.43 dB	53.59 dB	78.82 dB
9	14.39 dB	34.45 dB	32.43 dB	49.09 dB	35.51 dB	61.70 dB
10	10.29 dB	42.58 dB	54.72 dB	37.01 dB	52.16 dB	66.09 dB
11	19.66 dB	50.97 dB	53.73 dB	37.35 dB	45.99 dB	58.63 dB
12	22.46 dB	32.45 dB	27.20 dB	40.51 dB	32.97 dB	81.20 dB
13	20.90 dB	62.07 dB	55.33 dB	53.17 dB	42.30 dB	87.79 dB
14	20.84 dB	61.12 dB	55.94 dB	32.48 dB	43.99 dB	80.64 dB
15	15.42 dB	36.95 dB	26.76 dB	42.37 dB	28.99 dB	59.85 dB
Average	17.55 dB	44.31 dB	42.70 dB	41.32 dB	42.36 dB	70.01 dB

**Table 2:** Parts of characteristic vectors of defects.

Broken Wires	*A*	*R*	*E*	$\phi_{1}$	$\phi_{2}$	$\phi_{3}$	$\phi_{4}$	$\phi_{5}$	$\phi_{6}$	
1	2.37 × 10^4^	0.549	0.404	6.664	28.63	35.55	39.67	80.75	54.36	77.35
2	3.55 × 10^4^	0.702	0.222	6.664	29.53	39.09	35.19	72.91	49.96	72.54
3	4.72 × 10^4^	0.744	0.262	6.665	26.87	33.75	35.69	71.36	49.62	74.19
4	3.08 × 10^4^	0.763	0.412	6.667	26.75	33.39	33.40	69.74	47.93	67.98
5	5.82 × 10^4^	0.609	0.568	6.668	25.43	33.08	33.34	66.60	46.42	68.99
7	9.74 × 10^4^	0.732	0.727	6.669	26.89	33.73	31.82	66.01	47.26	64.72
